# Jia-ga-song-tang protection against alcoholic liver and intestinal damage

**DOI:** 10.3389/fphar.2022.981706

**Published:** 2022-09-26

**Authors:** Jiamin Fang, Yuhuan Wu, Changlian Gan, Shufang Ruan, Xiaoliang He, Bixia Wang, Ying Wang, Jingtao Yu, Chuanlan Sang, Dawa Zeren, Tianqin Xiong

**Affiliations:** ^1^ School of Pharmaceutical Sciences, Guangzhou University of Chinese Medicine, Guangzhou, China; ^2^ Laboratory of Experimental Animal, Guangzhou University of Chinese Medicine, Guangzhou, China; ^3^ Research Department, University of Tibetan Medicine, Lhasa, China

**Keywords:** alcoholic liver disease, jia-ga-song-tang, network pharmacology, proteomics, liver protection

## Abstract

Gut-liver axis and cellular homeostasis play key roles in alcohol liver disease (ALD). Nuclear factor (erythroid-derived 2)-like 2 (Nrf2) is a stress-sensitive guarantor of cellular homeostasis. We investigated whether the beneficial effects and underlying mechanisms of Jia-ga-song Tang (JGST) against ALD were associated with gut-liver axis and cellular homeostasis. A predictive network depicting the relationship between Jia-Ga-Song-Tang (JGST) and alcoholic liver disease (ALD) was designed by Network pharmacology. Next, 5% v/v Lieber-DeCarli alcohol liquid diet was used to establish the ALD. JGST protected the liver damage, repaired the intestines to alleviate the Two-hit on the liver, and balanced the cellular homeostasis. It was manifested in repairing the liver and intestinal pathological structure, reducing serum ALT, AST, and liver TG, TC, MDA, CAT, and increasing liver GSH, and intestine GSH-Px. JGST mainly inhibited the liver mRNA levels of HO-1, NQO1, GCLC, FASN, and PPARα and activated the intestinal mRNA levels of HO-1 and NQO1, while inhibiting the liver protein levels of HO-1, NQO1. Furthermore, LPS and LBP in the plasma and the expression of inflammatory factors such as IL-1β, TNF-α, IL-6, TGFβ1, CD14, and Myd88 were reduced after treatment to prove that JGST protects the liver from Two-hit. Ethanol was used to intervene in HepG2 and IEC-6 to establish an ALD cell model and treated by Germacrone, ML385, and TBHQ. repaired the intestinal barrier, and inhibited Nrf2 in IEC-6, but protect the HepG2 by activating Nrf2 to balance cellular homeostasis. Our results reinforce that JGST provides an effective protective method for alcoholic liver disease (ALD) by regulating Gut-liver axis and cellular homeostasis.

## Introduction

Excessive alcohol consumption can lead to a series of alcohol-related liver diseases, collectively known as alcoholic liver diseases (ALD). Alcoholic fatty liver or steatosis is seen early in the histological part, develops into alcoholic hepatitis, and could eventually evolve into end-stage liver diseases (liver failure, cirrhosis, and liver cancer) (Hyun et al., 2021). Studies have found that ALD is a common type of liver disease affecting millions of people worldwide (Han et al., 2021a). Clinically, the counter-measures for alcoholic liver injury are complete abstinence from alcohol along with nutritional support. However, there are no recognized specific drugs for ALD. Clinical medications include disulfiram and corticosteroids; however, those interventions have adverse effects. Therefore, there is a need to find drugs with good efficacy and low side effects to treat alcohol-induced liver toxicity (Liu et al., 2021).

The ALD mechanism is complex. Excessive ethanol, high acetaldehyde, and reactive oxygen species (ROS) produced by alcohol metabolism are the major causes of liver damage (Seitz and Stickel, 2006; Seitz et al., 2018). Excessive accumulation of ROS will lead to oxidative damage, lipid peroxidation, DNA mutation, and cell membrane damage. Reactive oxygen species (ROS) as pleiotropic physiological signaling agents can regulate Nrf2 signal pathway, and the activation of Nrf2 will lead to the activation of downstream HO-1, NQO1, and so on (Sies and Jones, 2020). Furthermore, it has long been established that the gut-liver axis and alcohol-induced increased alcohol-induced gut permeability are important in the pathogenesis of ALD (Szabo et al., 2022). The induction of alcohol metabolites leads to the destruction of the intestinal barrier. The enterocyte regulates the function of barrier and intercellular transport mainly through tight junction (TJ) and adhesive junction (AJ) (Lopetuso et al., 2015; Wells et al., 2017). Tight junctions mainly include transmembrane proteins and cytoplasmic actin-binding proteins. Transmembrane proteins are claudins, occludin and JAM, which are responsible for establishing cell-cell contact in the intercellular space, cytoplasmic actin-binding protein is mainly ZO (including ZO-1, ZO-2, ZO-3), which acts as the link of actin cytoskeleton (Campbell et al., 2017). The adhesion junction, located below the tight junction, contains two subunits: Cadherins and Nectins, AJ mainly mediates the adhesion between adjacent cells (Campbell et al., 2017). TJ and AJ are connected by ZO-1 to form a stable intercellular connection (Campbell et al., 2017). Bacteria and LPS (Lipopolysaccharide) enter the circulation through the damaged barrier, and the escaped LPS enters the hepatic portal vein through the ileum and is recognized by TLR4+ macrophages. TLR4 depends on LBP shuttling to CD14, CD14 to specifically bind to LPS and interact with TLR4 and Myd88 to regulate inflammatory factors and aggravate liver injury (Han et al., 2021b). Inflammation was a central element in the development of liver injury and a dynamic process of chronic liver disease. And the Two-hit to the liver is also an important cause of alcoholic liver injury (Cho and Song, 2018). Therefore, oxidative stress inhibition and intestinal barrier repair play an important role in ALD treatment.

Nuclear factor erythroid two like 2 (NFE2L2), also called Nrf2, is a transcription factor and the master regulator of cell homeostasis, which can regulate the expression of more than 200 genes involved in cell protection including heme oxygenase 1 (HMOX1), superoxide dismutase 1 (SOD1) and Recombinant NADH Dehydrogenase, Quinone 1(NQO1) (Salehabadi et al., 2022). Nrf2-mediated pathway is increasingly proposed as an approach to prevent or treat disease. We also found that JGST (Jia-Ga-Song-Tang) promotes Nrf2 into nuclear equilibrium oxidation (Chen et al., 2020).

Due to the Tibet terrain and climate features, the alpine and cold environment have encouraged a local drinking habit and a succulent diet, leading to a high incidence of liver disease. Thus, for over 2,000 years, Tibetan medicine has had a unique understanding of liver disease treatment (Baima, 2014). JGST, also known as SanWeiGanJiang San (ginger, cardamom, nutmeg), is a traditional prescription used in Tibetan medicine to treat liver diseases and is composed of a mixture of medicine and food homology of traditional Chinese medicine.

Previous HPLC study has determined the chemical constituents and fingerprints for JGST, and the JGST fingerprints were analyzed to identify 15 peaks, two of which were 6-gingerol and dihydride-isoeugenol. The volatile chemical components from JGST were analyzed by Gas chromatography-mass spectrometry (GC-MS) to provide evidence for the scientific and the ratio of JGST, and the JGST optimal extraction process was obtained through the orthogonal experiment (Tan et al., 2014; Xiong et al., 2013a; Zhao et al., 2014). We previously reported that JGST reduces the ALT and AST abnormal elevation in various acute or chronic models of liver injury. This prescription can also repair the pathological damage to liver and the intestinal barrier. JGST could regulate the bile acid enterohepatic circulation thereby maintaining the gut-liver axis homeostasis (Fu et al., 2014; Liao et al., 2015; Wu et al., 2016; Xiong et al., 2013). JGST plays a role in protecting the liver by promoting the Nrf2 entry into the nucleus and the Bach1 exit from the nucleus, regulating the downstream antioxidant proteins HO-1 and NQO1 (Chen et al., 2020). However, the JGST therapeutic effect on alcoholic liver injury and its underlying mechanism remains unclear. Therefore, this study aims to define the JGST anti-ALD effect and clarify its mechanism.

In this study, after preliminary exploration of the effect of JGST against ALD through network pharmacology, a 5% v/v Lieber-DeCarli alcohol liquid diet was used to establish alcoholic liver injury and observe the effect of JGST on injury and the subsequent effect of JGST on intestinal and liver injury. Furthermore, through the ALD liver proteomics and ALD cell model, an attempt was performed to clarify the regulatory mechanism of JGST and provide a reference for clinical medication for alcoholic liver injury treatment.

## Materials and methods

### Animals

80 male C57BL/6 J mice were purchased from the Si Pei Fu Bio-Technology Co., Ltd. (Beijing, China) and acclimated for 7 days in a controlled environment room (12-h dark/light cycle, temperature: 25°C, humidity:40–70% with free access to water and food). The study was approved by the Experimental Animal Management and Ethics Committee of the Guangzhou University of Chinese Medicine. (Guangzhou, China, certificate number: No.00247613, Ethics Certificate number: ZYD-2020-072).

### Reagents

JGST consists of Dried rhizome of Zingiber officinale Roscoe (Gang Jiang (GJ)), and Dry ripe fruit of Amomum compactum Sol. Ex Maton (Dou Kou (DK)), Dried kernel of Myristica fragrans Houtt (Rou Doukou (RDK)). All herbs were purchased from Guangdong Province Traditional Chinese Medicine Hospital, pounded into powder, sieved through a NO.6 standard sieve, and mixed in a 6:5:4 (GJ: DK: RDK) ratio (Xiong et al., 2013a). The Lieber-DeCarli alcoholic liquid diet was purchased from Trophic Animal Feed High-tech Co., Ltd., China, and silymarin capsules were purchased from MADAUS GMBH (Koeln, Germany); THBQ and ML385 were purchased from MedChemExpress (shanghai, China). ALT、AST、MDA、GSH-Px assay kits were purchased from Nanjing Jiancheng Bioengineering Institute (Nanjing, China). Fetal bovine serum was obtained from Gibco (Gibco, Waltman, United States). Detailed information are listed in Supplementary Table S2.

### Pharmacology network construction and analysis

Briefly, 1) In the HREB database (Health Research Ethics Board HREB (http://herb.ac.cn/)), the key words GJ, DK, and RDK were used to search and obtain the relevant components information of traditional Chinese medicine. And the 2D structures information of components found earlier were used to obtain potential targets from the database: PubChem (https://pubchem.ncbi.nlm.nih.gov/). The Gene name of the components were collected in the SwissTargetPrediction database (http://www.swisstargetprediction.ch/), and the all targets were selected to integrate the target set of the components. After this step, the components of JGST and the corresponding gene names are obtained. Then, the key word “alcohol liver disease” was used to search the ALD-related targets from the DisGeNET (http://www.disgenet.org/), the OMIM (http://omim.org/), GeneCards (https://www.genecards.org/), DRUGBANK (https://www.drugbank.com/), and TTD (http://db.idrblab.net/ttd/). 2) The overlapping targets of component and ALD were inputted into the STRING database (https://string-db.org/), the species was set to “Homosapiens”, the lowest interaction threshold “Medium confidence” was set to 0.400, and other parameters were defaulted without processing to obtain the interaction relationship of the targets. Then the target interaction network (PPI) is made by Cytoscape3.8.0, and the NetworkAnalyzer in it is used for network analysis, and the PPI is made by adjusting the size of nodes in the network according to the degree of connectivity. And Gene Annotation & Analysis Resource was used to analyze the GO function and KEGG pathway enrichment of core targets. Cytoscape 3.8.0 was used for visualization operation. 3) Analyse the results according to the node degree and *p* values.

### Animal treatment

After adaptation to the control liquid diet for 3 days. All mice were divided into two groups: one group received only control liquid diet, and the other received alcoholic liquid diet from Day 4 to Day11 (2 days of 1.6% ethanol, 2 days of 2.5%, 3 days of 3.3%). Then, this group was further divided into five groups of, Model group (normal saline 0.1 ml/10 g), Silymarin positive group (54.6 mg/kg), JGST-L (0.17 g/kg), JGST-M(0.33 g/kg), and JGST-H (0.66 g/kg).

**FIGURE 1:**
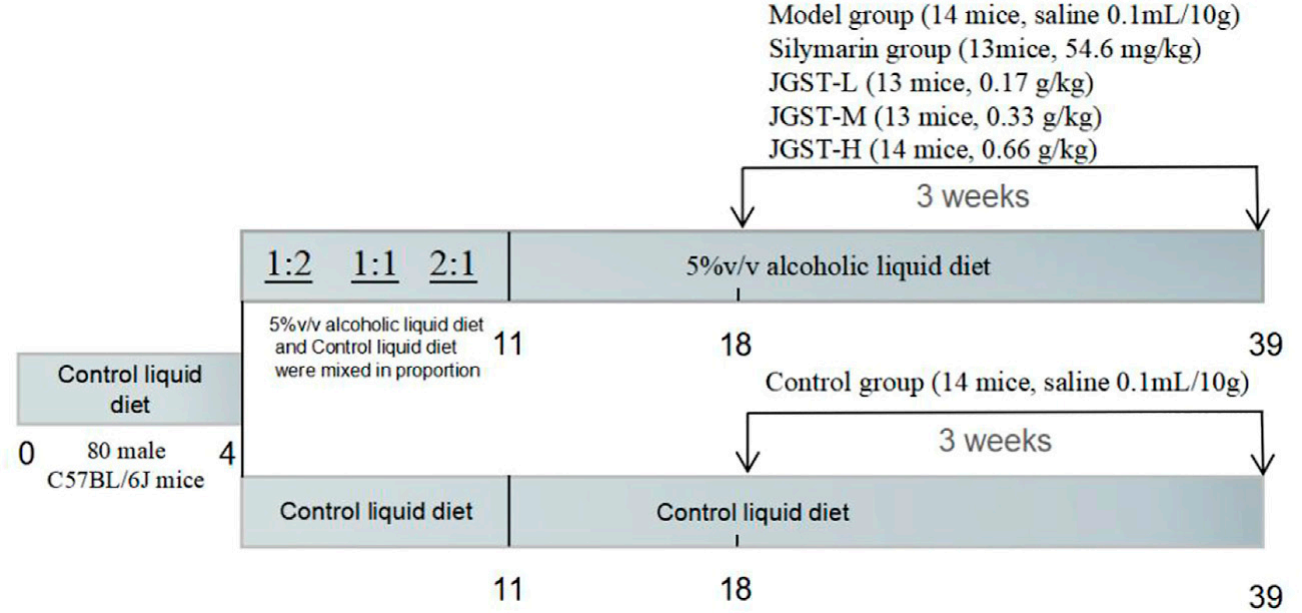
Overview of the alcohol induced liver injury mouse model procedure.

Especially, during the entire period, the normal control group and the rest of the groups were fed isocaloric feeding. Detailed information are listed (Figure 1).

Fresh blood samples were collected. The serum was then centrifuged at 3,000 rpm for 10 min at 4°C after incubation at 25°C for 2 h. Plasma was collected by a tube attached with Ethylenediaminetetraacetic acid (EDTA) anticoagulant and centrifuged at 3,000rpm for 10 min at 4°C. The supernatant was collected for further analysis. The cervical dislocation was used for animal execution, and the liver and intestine of the mice were stripped, weighed, and partly fixed in 4% paraformaldehyde for histopathological analysis. The other samples were stored at -80°C for biochemical analysis and western blotting (Ahmed et al., 2015; Ebeid et al., 2015).

### Biochemical analysis and enzyme-linked immunosorbent assay

Serum AST and ALT levels were detected using biochemical kits. The contents of TG, TC, MDA, GSH, CAT, and GSH-PX in the liver or intestinal homogenate (10%) were measured using biochemical kits (Bioengineering Institute, Nanjing, China) according to the manufacturer’s instructions. In addition, the levels of Lipopolysaccharide (LPS) and Lipopolysaccharide-binding protein (LBP) in the plasma were analyzed using an ELISA kit (CLOUD-CLONE CORP, United States). According to the manufacturer`s instructions, the expression of IL-1β in the liver was detected using the Elisa kit (Thermo Fisher Scientific).

### Histological analysis

The liver and intestine tissues were fixed in 4% paraformaldehyde, then gradient dehydrated, embedded in paraffin wax, and stored at room temperature (20–25°C). Next, the mice’s liver and intestine tissues were sliced and dewaxed before staining.

The liver stored at -80°C was embedded in a Microtome Cryostat (Leica Biosystems, Nubloch, Germany) with Optimal Cutting Temperature compound (OCT) at -20°C, then sliced and fixed rinsed (10% neutral formalin, isopropanol), and stained with Oil Red O staining in the dark. Haematoxylin-eosin (HE) and Oil Red O staining were performed according to standard protocols.

The pathological changes and lipid droplets in the sections were observed under a microscope (BX53, Olympus Corporation, Tokyo, Japan), and images were captured.

### Cell culture and treatment

Intestinal crypt epithelial cells (IEC-6) and HepG2 cells were obtained from the Cell Resource Center, Peking Union Medical College (which is the headquarters of the National Infrastructure of Cell Line Resource, NSTI). In cell culture flasks, cells were cultivated in Dulbecco’s Modified Eagle Medium basic (Gibco, 1×) with 10% FBS (Gibco) and 1% penicillin-streptomycin solution (Gibco) in a humidified incubator of 5% CO_2_ at 37°C.

HepG2 cells were divided into groups: the control group, the model group (ethanol 200 mmol/L), the ML385 positive group (5 μmol/L), the Germacrone group (GM-L: 0.5 μmol/L, GM-M: 1 μmol/L, GM-H: 2 μmol/L), THBQ group (10 μmol/L), THBQ + Germacrone group (0.5 μmol/L, 1 μmol/L).

IEC-6 cells were divided into groups: the control group, the model group (ethanol 500 mmol/L), the THBQ positive group (5 μmol/L), the Germacrone group (GM-L: 1 μmol/L, GM-M: 2 μmol/L, GM-H: 4 μmol/L).

Germacrone, ML385, and tert-Butylhydroquinone (THBQ) were pre-administrated and lasts giving a total of 24 h of administering. HepG2 cells were treated with 200 mM ethanol for 2h, while IEC-6 cells with 500 mM ethanol for 18 h. RT-qPCR was used to observe the cell expression of the gene, and the western blot was used to detect the expression of the protein.

### Real-time qPCR analysis and western blot

The liver and ileum were stored in RNA protection solutions at -20 °C. First, total RNA was extracted from both *in vivo* and vitro using the RNA extraction kit (Beyotime Biotech Inc., Shanghai, China), and the RNA concentration was determined using NanoDrop (Gibriel et al., 2013; Gibriel et al., 2019). Next, we followed the instructions for the cDNA synthesis kit (PrimeScript™ RT Master Mix (Perfect Real Time)) for reverse transcription (Takara Biomedical Technology Co. Ltd., Beijing, Japan). qRT-PCR was performed with the TB Green® Premix Ex Taq™ II (Tli RNaseH Plus) (Takara Biomedical Technology Co. Ltd., Beijing, Japan). The mRNA expression of NQO1, HO-1, GCLC, PPARα, FASN, IL-1β, TNF-α, IL-6, TGF-β1, ZO-1, Occludin, Claudin2, and GAPDH were analyzed by qPCR in a 7,500 Real-Time PCR System and the PCR primer sequences are listed in Tables 1–Tables 3. Results were quantified by the 2^−ΔΔCt^ method relative to the housekeeping gene GAPDH (Gibriel et al., 2020; Yasser et al., 2021).

**TABLE 1:** The PCR primers sequences for RT-qPCR(Mouse).

Gene	Forward	Reverse	Gene id
Pparα	GAG​GTG​CAA​GAT​TCA​GAA​GAA​G	GAA​TCT​TTC​AGG​TCG​TGT​TCA​C	ID: 19013
Fasn	TAA​AGC​ATG​ACC​TCG​TGA​TGA​A	GAA​GTT​CAG​TGA​GGC​GTA​GTA​G	ID: 14104
Ho-1	TCC​TTG​TAC​CAT​ATC​TAC​ACG​G	GAG​ACG​CTT​TAC​ATA​GTG​CTG​T	ID: 15368
Nqo1	GAA​GAC​ATC​ATT​CAA​CTA​CGC​C	GAG​ATG​ACT​CGG​AAG​GAT​ACT​G	ID: 18104
Gclc	CTA​TCT​GCC​CAA​TTG​TTA​TGG​C	CCT​CCC​GTG​TTC​TAT​CAT​CTA​C	ID: 14629
Zo-1	CTG​GTG​AAG​TCT​CGG​AAA​AAT​G	CAT​CTC​TTG​CTG​CCA​AAC​TAT​C	ID: 21872
Occludin	TGC​TTC​ATC​GCT​TCC​TTA​GTA​A	GGG​TTC​ACT​CCC​ATT​ATG​TAC​A	ID: 18260
Claudin2	GGT​TCC​TGA​CAG​CAT​GAA​ATT​T	GCC​ATC​ATA​GTA​GTT​GGT​ACG​A	ID: 12738
Il-1β	TCG​CAG​CAG​CAC​ATC​AAC​AAG​AG	AGG​TCC​ACG​GGA​AAG​ACA​CAG​G	ID: 16176
Tnf-α	ATG​TCT​CAG​CCT​CTT​CTC​ATT​C	GCT​TGT​CAC​TCG​AAT​TTT​GAG​A	ID: 21926
Il-6	CTC​CCA​ACA​GAC​CTG​TCT​ATA​C	CCA​TTG​CAC​AAC​TCT​TTT​CTC​A	ID: 16193
Tgf-β1	CCA​GAT​CCT​GTC​CAA​ACT​AAG​G	CTC​TTT​AGC​ATA​GTA​GTC​CGC​T	ID: 21803
Gapdh	GGT​TGT​CTC​CTG​CGA​CTT​CA	TGG​TCC​AGG​GTT​TCT​TAC​TCC	ID: 14433

**TABLE 2:** The PCR primers sequences for RT-qPCR(Human).

Gene	Forward	Reverse	Gene id
HO-1	AAG​AGG​CCA​AGA​CTG​CGT​TC	GTA​AGG​ACC​CAT​CGG​AGA​AGC	ID: 3162
NQO1	GAA​GAC​ATC​ATT​CAA​CTA​CGC​C	GAG​ATG​ACT​CGG​AAG​GAT​ACT​G	ID: 1728
GAPDH	CAG​GAG​GCA​TTG​CTG​ATG​AT	GAAGGCTGGGGCTCATTT	ID: 2597

**TABLE 3:** The PCR primers sequences for RT-qPCR(Rat).

Gene	Forward	Reverse	Gene id
HO-1	CAG​GTG​TCC​AGG​GAA​GGC​TTT​AAG	TGG​GTT​CTG​CTT​GTT​TCG​CTC​TAT​C	ID: 24451
NQO1	AGG​ATG​GGA​GGT​GGT​CGA​ATC​TG	GCC​TTC​CTT​ATA​CGC​CAG​AGA​TGA​C	ID: 24314
ZO-1	CGC​AGC​CAG​TTC​AAA​CAA​AGT​TCC	GCA​ACA​TCA​GCA​ATC​GGT​CCA​AAG	ID: 292994
Occludin	CAA​CGG​CAA​AGT​GAA​TGG​CAA​GAG	TCA​TCC​ACG​GAC​AAG​GTC​AGA​GG	ID: 83497
Gapdh	GAC​ATG​CCG​CCT​GGA​GAA​AC	AGC​CCA​GGA​TGC​CCT​TTA​GT	ID: 24383

The protein samples of liver and cells were extracted from the radioimmunoprecipitation assay (RIPA) buffer or Nuclear and Cytoplasmic Protein Extraction Kit (Beyotime Biotech Inc., Shanghai, China). The BCA kit was used to quantify the protein concentration. Next, 30 μg of protein and 20 μg of Nuclear protein were loaded for electrophoresis and then transferred to a Polyvinylidene fluoride (PVDF) membrane. After blocking with 5% skimmed milk powder for 1 h, the primary antibody was diluted proportionally, and the membrane was incubated overnight at 4 °C followed by incubation with a secondary antibody for 1 h. Finally, the bands were visualized with a Chemiluminescent and photographed. ImageJ (National Institutes of Health and the Laboratory for Optical and Computational Instrumentation, CA, United States) was used to analyze the average gray value of the target strip. Detailed information of antibodies and dilution ratio are listed in Supplementary Table S1.

### Proteomics analysis

Each group selected three biological replicates for proteomics analyses. The proteins in the liver were extracted sent to the Shanghai Meiji Biological Co., Ltd, and used for TMT proteomic detection. The data were analyzed on the Majorbio Cloud Platform (www.majorbio.com).

Protein identification: The RAW data files were analyzed using ProteomeDiscoverer (Thermo Scientific, Version 2.4) against Mus_musculus database (UniProt-taxonomy-10090-Mus musculus.20201214. fasta).

### Statistical analysis

After the Kolmogorov-Smirnov and Bartlett’s test, Analysis of Variance followed by Dunnett’s test was used to compare data that did not conform to the normal distribution or when variance was uneven, and the multiple *t*-test (Tukey’s test) was used to compare the two groups. Statistical significance was set at *p* < 0.05. GraphPad Prism 8.0 was used for drawing.

Proteomics: The MS/MS search criteria were as follows: Mass tolerance of 10 ppm for MS and 0.02 Da for MS/MS Tolorance, trypsin as the enzyme with two missed cleavage allowed, carbamidomethylation of cysteine, and the TMT of N- terminus and lysine side chains of peptides as fixed modification, and methionine oxidation as dynamic modifications, respectively. The false discovery rate (FDR) of peptide identification was set as FDR ≤0.01. A minimum of one unique peptide identification was used to support protein identification. The thresholds of fold change (>1.2 or <0.83) and *p*-value <0.05 were used to identify differentially expressed proteins (DEPs).

## Results

### Analysis of JGST against ALD by network pharmacology

**FIGURE 2:**
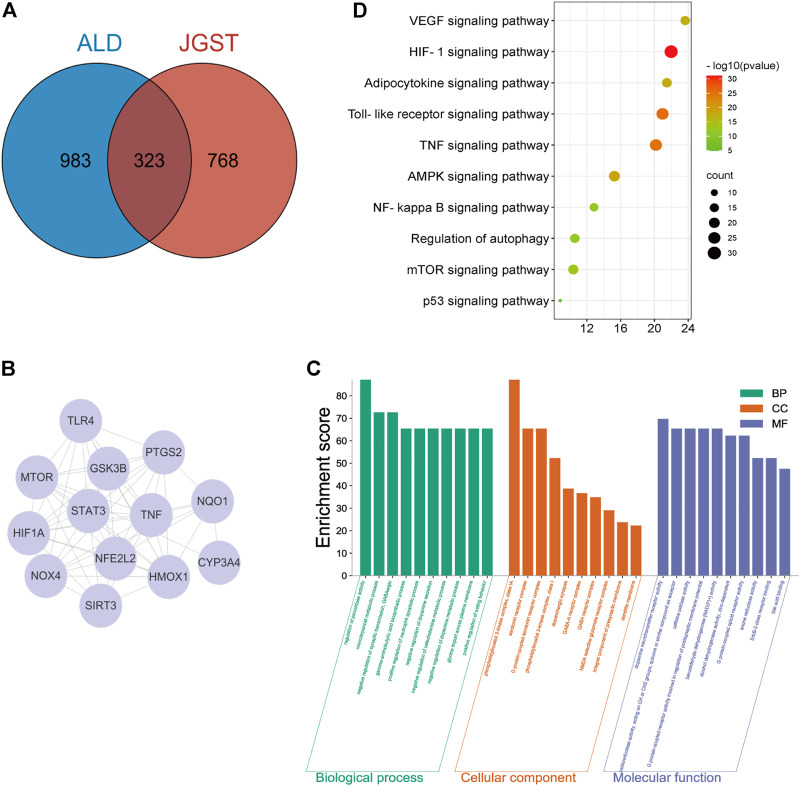
JGST on Chronic Alcoholic Liver Injury by network Pharmacology **(A)** JGST-Chronic ALcoholic Liver Injury Intersection Target Venn Diagram. **(B)** PPI network diagram of the core target. **(C)** GO enrichment analysis of Overlapping target. **(D)** KEGG Pathway enrichment analysis of overlapping targets.

The databases revealed 1,091 potential targets of GJ, DK, and RDK. Analysis of data identified 1,306 targets associated with ALD. Finally, there were 323 overlapping targets (Figure 2A). A PPI of 323 overlapping targets comprised 6,304 edges. The core targets were extracted and analyzed, and it was found that the core targets included Tumor necrosis factor (TNF), Toll-likereceptor4 (TLR4), Heme oxygenase-1 (HO-1), and NAD(H)P-quinone-oxidoreductase -1 (NQO1) (Figure 2B).

The overlapping targets were used to identify Molecular Function (MF), Biological Process (BP), Cellular Component (CC) and pathways using GO and KEGG enrichment analyses, which revealed that BP mainly involves the regulation of peroxidase activity, inflammatory response, and glycine import across the plasma membrane. The CC mainly involves G protein-coupled serotonin receptor complex and presynaptic membrane. Molecular functions (MF) mainly involve oxidoreductase activity and alcohol dehydrogenase (NAD+) activity. In addition, KEGG enrichment analysis indicated that the overlapping targets enriched in glutathione, glycerolipid, fatty acid metabolism and degradation, and Toll-like receptor signaling pathway, indicating that JGST in chronic alcoholic liver injury may be related to the regulation of oxidative stress, fatty acid, and drug metabolism-P450 metabolic enzymes (Figures 2C,D).

### The effect of JGST on steatosis in the liver of ALD mice

**FIGURE 3:**
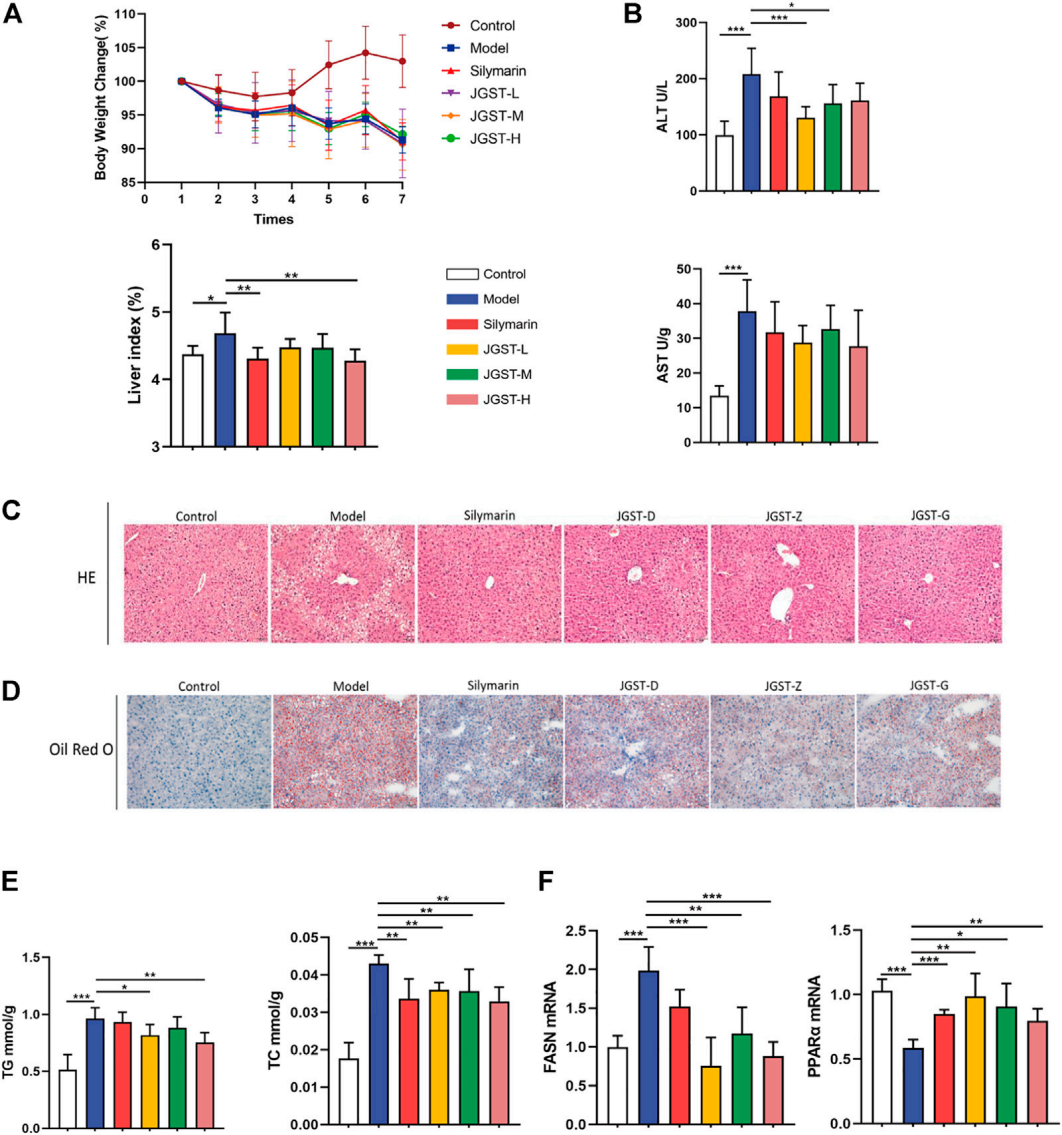
Liver protection effect for JGST against ALD induced mouse model. **(A)** Mouse body weight changes and liver index. **(B)** Liver function enzymes ALT and AST **(C)** Representative photomicrographs of liver tissue with H&E staining (x200, 50 p.m., n = 6). **(D)** Representative photomicrographs of liver tissue with oil red O staining (x 200, 50 p.m., n = 6). **(E)** Liver level of cytoplasmic TG and TC. **(F)** mRNA expression of PPARa and FASN in liver tissues. Control: normal control group, Model: model group, Silymarin: silymarin-positive group, JGST-L: the low-dose group of JGST; JGST-M: the medium-dose group of JGST; JGST-H: high-dose of JGST. Data were presented as Meant ± SEM, *p < 0.05, “*p* < 0.01, ***p < 0.00I, n = 7.

First, the establishment of a stable ALD model is the basis for studying the effect of JGST against Alcohol-Induced liver disease. HE staining showed that compared with the control group, EtOH-fed mice showed the appearance of fat vacuoles and destruction of liver structure. The silymarin and JGST improve the number and size of fat vacuoles. In addition, Oil red O staining showed a large number of lipid droplets were increased in liver tissue in the model group, and both silymarin and JGST reduced the lipid droplets in the liver. Our results showed that the mice’s body weight in the normal group increased overall. On the contrary, bodyweight gradually decreased daily in the model group, even after treating with silymarin and JGST (Figure 3A), and the loss of weight after Lieber-DeCarli alcoholic liquid diet was also consistent with the characteristics of the literature. And also confirmed that JGST improved liver injury of ALD mice including decreased liver index (Figure 3A), serum ALT (Figure 3B), liver TG, and liver TC (Figure 3E). Among them JGST-L and JGST-M showed significance for ALT, while AST showed a downward trend, but there was no significant. Alcohol causes lipid accumulation. As we discussed earlier, JGST significantly reduced liver triglyceride (TG) and total cholesterol (TC). Compared with the normal group, mRNA expression of the liver peroxisome proliferator-activated receptor *a* (PPARα) was significantly decreased in the model group and significantly increased after treatment. However, mRNA expression of fatty acid synthase (FASN) expression in the liver had contrasting results to that of PPARα. Thus, JGST improves liver lipid accumulation caused by chronic alcohol consumption.

### The effect of JGST on oxidative stress in the liver of ALD mice

**FIGURE 4:**
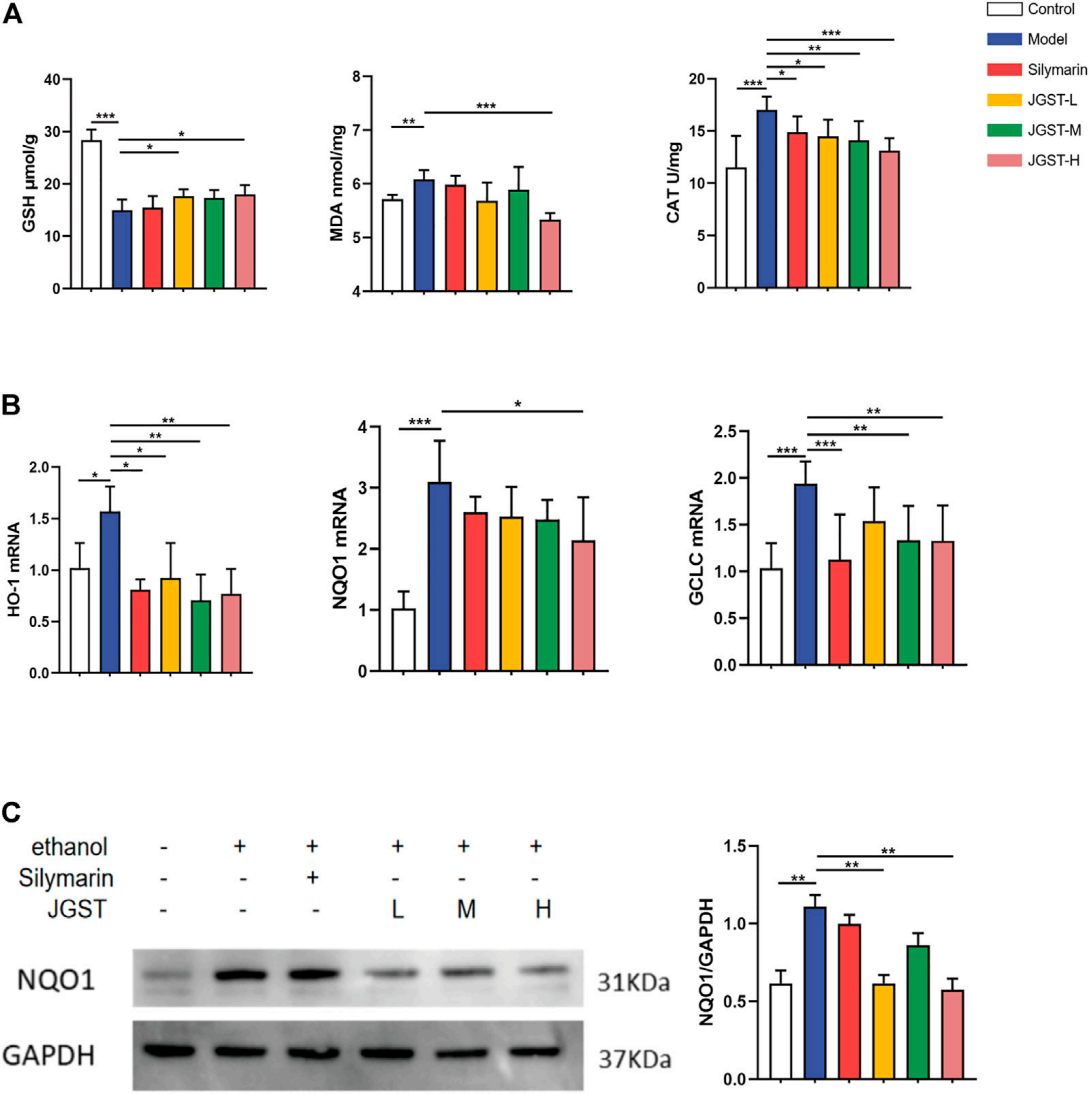
The effect of IGST on oxidative stress in the AI,D mice liver. **(A)** Liver level of GSH, MDA and CAT. **(B)** mRNA expression of HO-1, NQO1 and GCLC in liver tissues. **(C)** The protein expression of NQO1 in liver tissues. Data were presented as Meant ± SEM, *p < 0.05, **p < 0.01, ***p < 0.001, n = 7.

The damage caused by alcohol is probably due to oxidative stress. The results showed that JGST significantly reduced antioxidant enzyme CAT and JGST-H significantly decreased the lipid peroxidation end product malondialdehyde (MDA) (Figure 4A). JGST-L and JGST-H significantly increased the content of glutathione (GSH) in the liver (Figure 4A). In addition, JGST significantly reduced the mRNA expression of HO-1, and GCLC in the liver. JGST-H was shown with signifificant changes in NQO1 at the transcriptional level, (Figure 4B). Also, the protein expression of NQO1 showed a similar trend as the mRNA expression from WB, JGST-H decreased the protein and gene expression of NQO1. (Figure 4C). Overall, JGST affected the internal redox balance of the liver.

### The effect of JGST on oxidative stress and steatosis in the liver of ALD mice by hepatic proteomics

**FIGURE 5:**
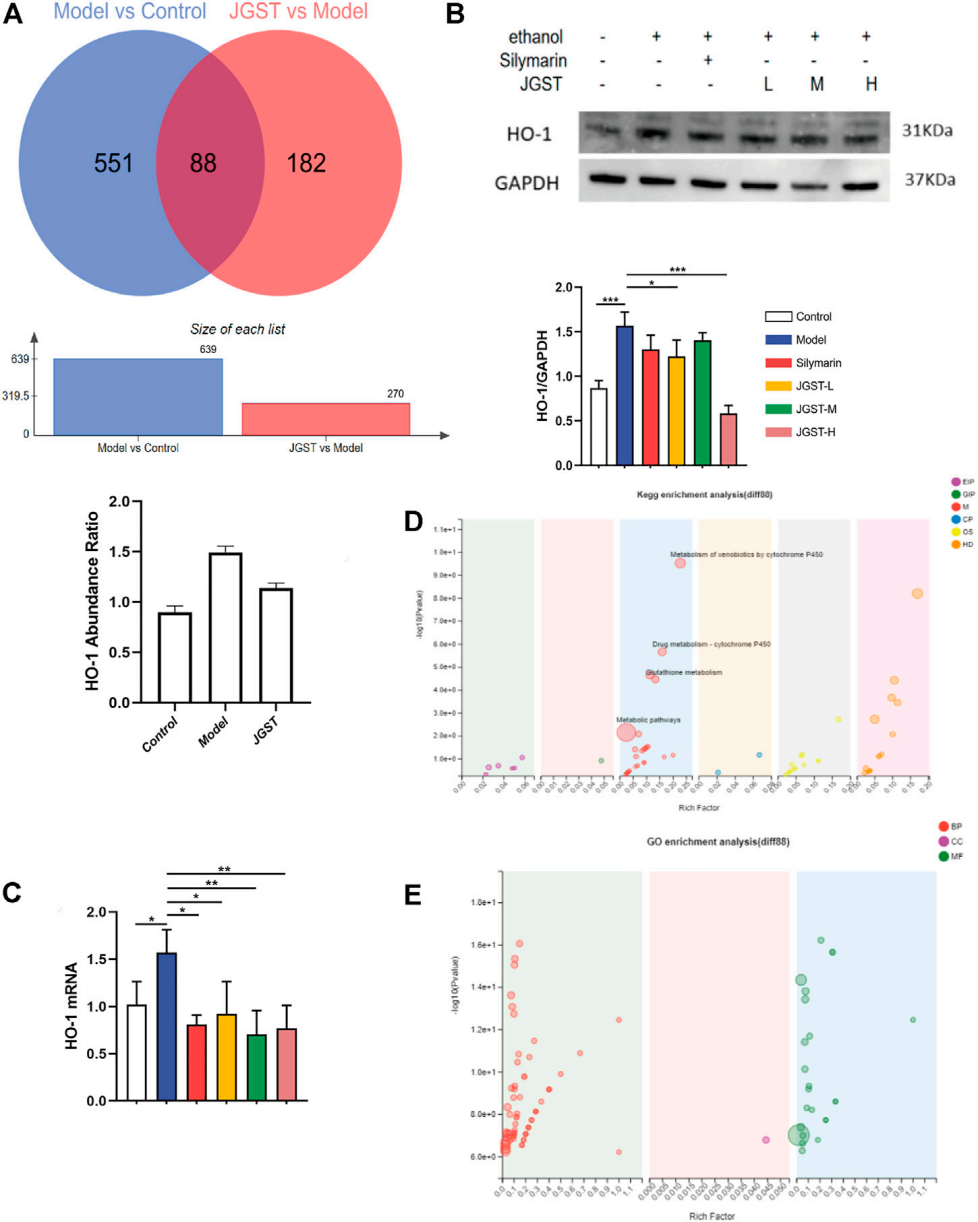
Differential proteins and overlapping protein enrichment analysis in the liver of ALD mice. **(A)** differential protein analysis in proteomics. **(B)** The protein expression of HO-1 in liver tissue. **(C)** GO enrichment analysis. **(D)** KEGG enrichment analysis. **(E)** GO enrichment analysis. Data were presented as Meant ± SEM, *p < 0.05, **p < 0.01, ***p < 0.001, n = 3.

The effects of JGST in the liver of ALD mice were investigated by TMT proteomic analysis. Furthermore, to identify the proteins altered significantly when giving alcohol or JGST changes, a fold change cut-off of >1.2 for up-regulation or <0.833 for down-regulation with a *p*-value cut-off of 0.1 was set. There were 639 significantly different proteins between the normal group and model group. Compared with the model group, the JGST group had 270 significantly different proteins, and 88 were overlapping (Figure 5A).

HO-1 was found to have a significant increase in abundance after alcohol administration and a significant decrease after administration. The trend in WB and RT-qPCR was similar to that of proteomics (Figure 5B).

In order to identify the functions of overlapping proteins, GO enrichment analysis and KEGG enrichment analysis were performed. Our results showed significant enrichment in the peroxidase activity, glutathione, and drug metabolism pathways. Thus, it showed that JGST in chronic ALD might involve the oxidation and inflammation pathway and fatty acid metabolism. Furthermore, these results were consistent with that of the network pharmacology, proving that the therapeutic effect of JGST on chronic ALD involves oxidative stress, fatty acid metabolism, and other pathways.

### The effect of JGST on the intestine in mice with ALD

Compared with the control group, the intestinal villi were arranged irregularly, the length was shortened, and the gap between the villi and basal layer increased. However, the conditions improved after treatment with silymarin and JGST.

**FIGURE 6:**
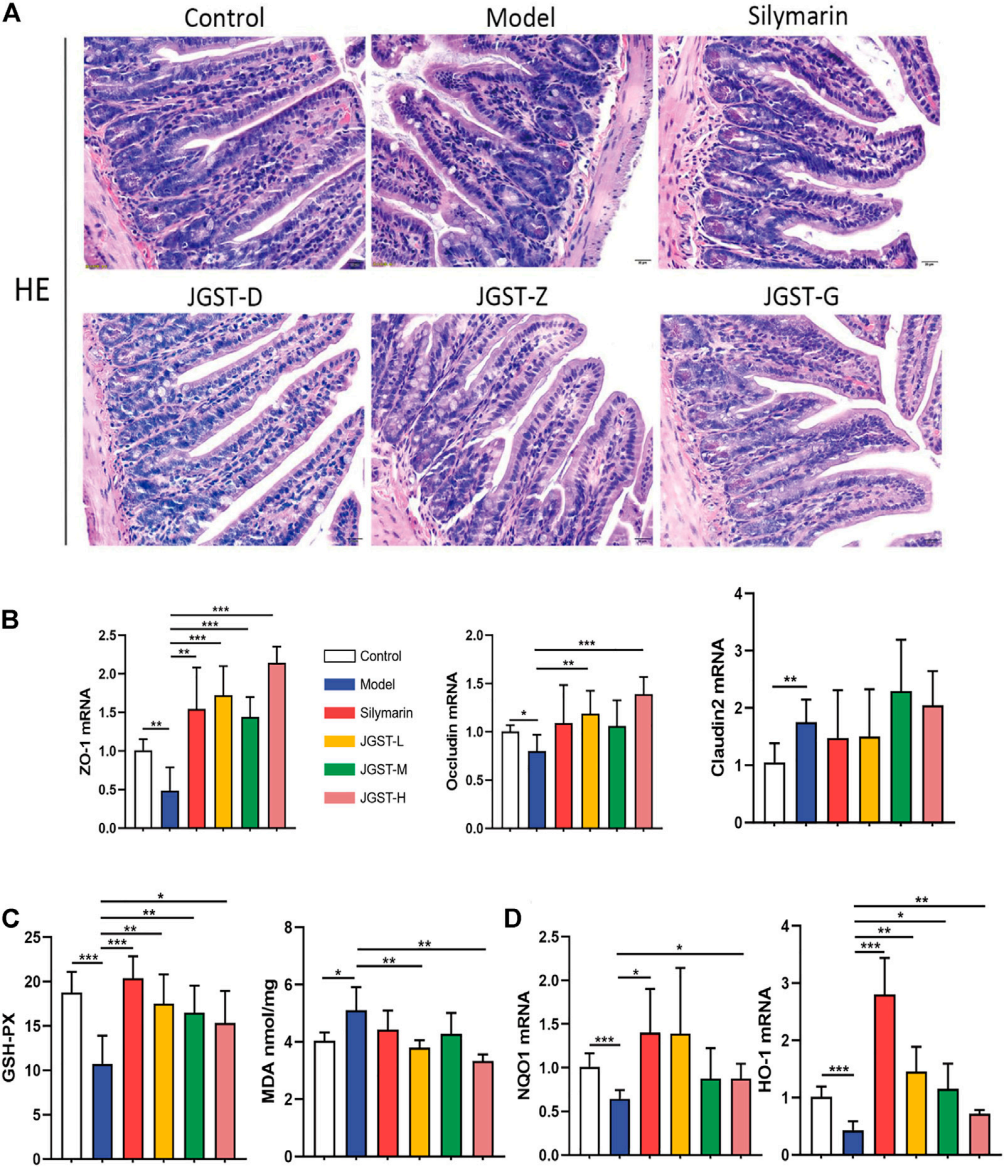
Protected effect of intestine damage of JOST on ALD. **(A)** Representative photomicrographs of intestine tissue with H&E staining (x200, 50 p.m., n = 6). **(B)** mRNA expression of ZO-1, Oecludin & Claudin2 in intestine tissues. **(C)** Intestine level of GSH-PX, MDA. **(D)** mRNA expression of NQO I &HO-1 in intestine tissues. Data wcrc presented as Meant ± SEM, *p.<0.05, **p < 0.01, ***p < 0.001, n = 7.

As shown in the pathology of intestinal tissue, alcohol leads to the destruction of the intestinal barrier. Furthermore, we found that JGST significantly increased the mRNA expression levels of intestinal tight junction proteins ZO-1 and Occludin (Figure 6B). In addition, the Claudin2 on the intestinal barrier was significantly higher in the model group than in the normal group (*p* < 0.01) but did not decrease after treatment. Thus, showing that JGST protects the intestinal barrier caused by alcohol.

We observe significant activation of internal redox balance, including, intestinal MDA, and increased glutathione peroxidase GSH-Px (Figure 6C). Treatment of JGST could reduce the MDA and GSH-Px. In addition, as shown in Figure 6D, JGST was inclined to significantly improve ALD by increasing the mRNA expression of the intestinal HO-1 and JGST-H signifificant changes in NQO1 at the transcriptional level. Thus, JGST balances oxidative stress to protect the intestines.

### Effect of JGST on inflammation in the liver of ALD mice

**FIGURE 7:**
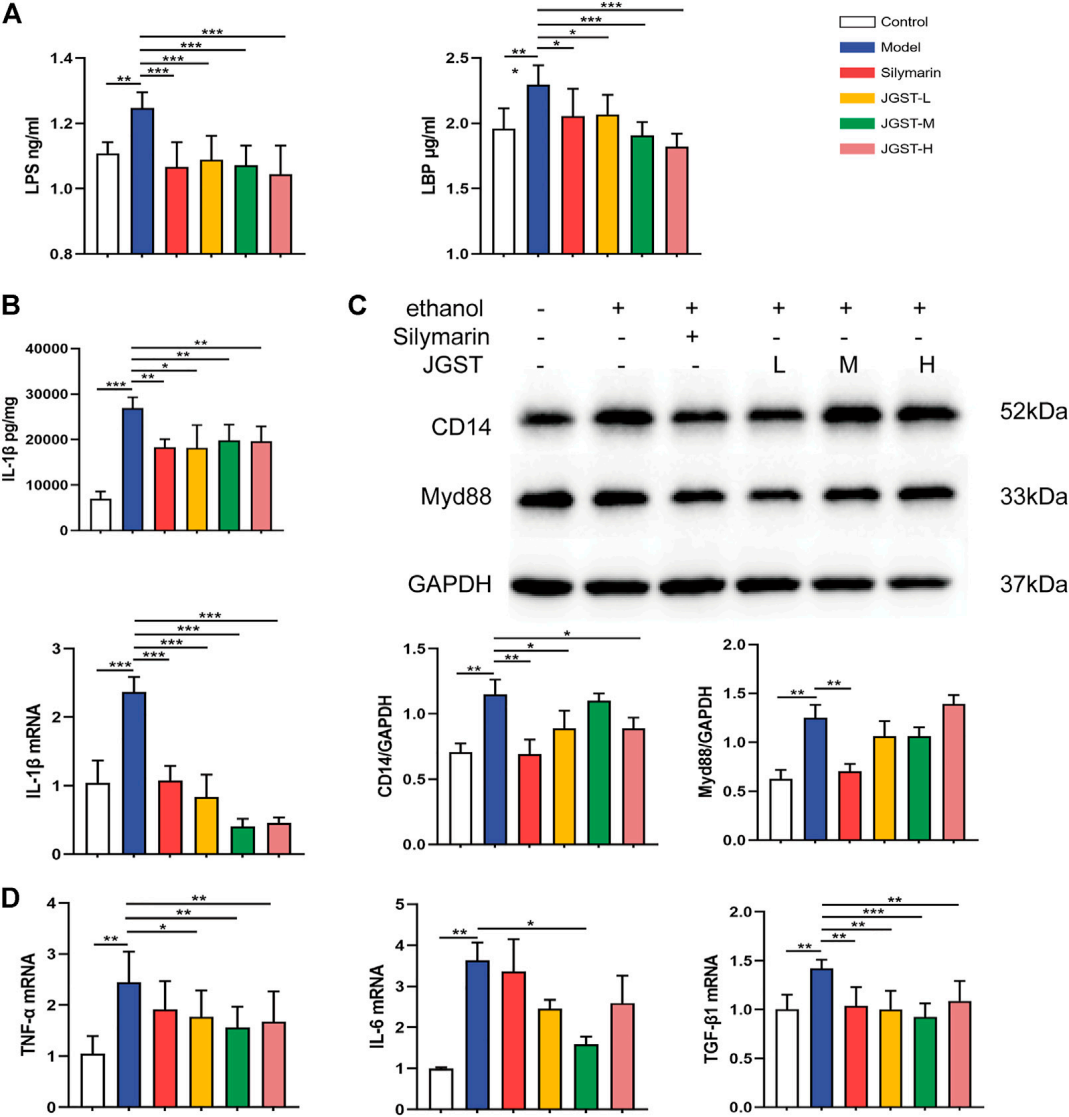
The effect of, IGST on inflammation in the liver of ALD mice. **(A)** Level of LPS and LBP in liver tissue. **(B)** Level and mRNA expression of IL-1β. **(C)** The protein expression of Myd88 and CD14 in liver tissues. **(D)** mRNA expression of TNF-a, TGF-β, and IL-6 in liver tissues. Data were presented as Meant ± SEM, *p < 0.05, **Pc0.01, ***p < 0.00 1, n = 7.

Alcohol leads to the destruction of the intestinal barrier, leading to LPS leakage and causing an inflammatory response. Furthermore, JGST significantly reduces the LPS and LBP content in the plasma induced by alcohol (Figure 7A).

The detection of inflammatory pathway proteins revealed that JGST significantly reduced the CD14 expression, and the Myd88 expression decreased. Reveals that JGST improved liver inflammation by reducing the inflammatory pathway proteins expression (Figure 7C).

Oxidative stress is closely related to inflammation, and ALD is usually accompanied by inflammation. The results showed that JGST significantly reduced IL-1β content in the liver (Figure 7B). Furthermore, RT-qPCR demonstrated that JGST significantly inhibited the mRNA expression of IL-1β, TNF-α, TGF-β1. And the expression of IL-6 was significantly inhibited by JGST-M. Therefore, JGST had effect on the internal anti-inflammatory system and protect the liver from inflammation.

### Effect of germacrone on ethanol *in vitro*

To further prove the effectiveness of Germacrone, the component of JGST, on the ALD, we further used the *in vitro* model. First, the best dosage of Germacrone was selected (0.5, 1, and 2 μM). At the same time, a liver injury cell model (200mM, 1h, ethanol) in HepG2, and an intestine barrier injury cell model (500mM, 18h, ethanol) in IEC-6 was established.

**FIGURE 8:**
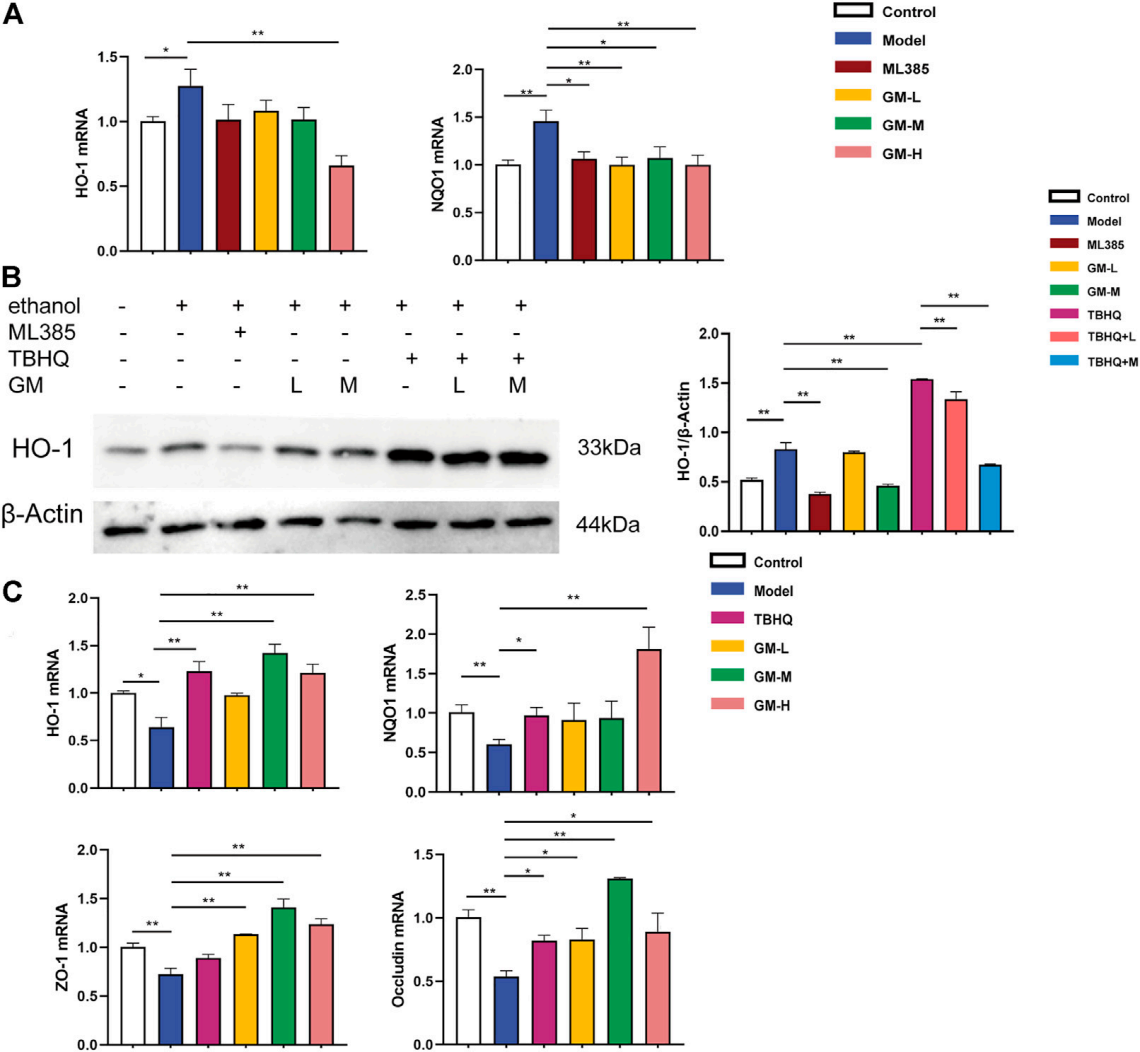
Protective effect of JGST against alcohol-induced liver injury in HepG2 and IEC-6 involved modulation of Nrf2. **(A)** mRNA expression of HO-1 and NQO1 in Hepg2. **(B)** The protein expression of HO-1 in HepG2. **(C)** mRNA expression of HO-I, NQOI, ZO-I&Occludin in IEC-6. Data were presented as Mean ± SEM, *p < 0.05, **.*p*.<0.01, ***p < 0.001, n = 3.

*In vivo*, our results showed that Nrf2 was activated in the livers of ALD mice. Meanwhile, HepG2 cells exhibited Nrf2 activation *in vitro* (ethanol, 200 mM). The protein expression of nuclear Nrf2 were Significantly upregulated in HepG2 induced by ethanol. Compared with the Model group, th protein expression of Nrf2 was signifificantly decreased in the GM-H group (Supplementary Figures S1A,B). The mRNA levels of HO-1 and NQO1 were substantially upregulated in HepG2 cells induced by ethanol. Upon treatment by Germacrone, the mRNA transcription of NQO1 were significantly downregulated and GM-H reduced the expression of HO-1, while the protein expression of HO-1 with a similar trend. Western blot further confirmed that Germacrone downregulated levels of HO-1 protein (Figures 8A,B).

Ethanol inhibited Nrf2 in IEC6 cells (ethanol, 500 mM). The mRNA expression of HO-1 and NQO1 were substantially downregulated in IEC-6 cells induced by ethanol. After treatment of Germacrone, the mRNA expression of HO-1 and NQO1 were significantly upregulated both in GM-H group. GM-M group also can upregulated the expression of HO-1. Meanwhile, as Figure 8C showed, the mRNA expression of intestinal tight junction proteins ZO-1 and Occludin were decreased in the model group, which means the intestinal barrier was injury. Further, we found that Germacrone significantly increased the mRNA expression of ZO-1 and Occludin in all three treatment groups.

## Discussion

The liver plays a key role in many physiological processes, including lipid and cholesterol homeostasis, immune responses, drug, nutrient, glucose metabolism, and other processes (Trefts et al., 2017). Moreover, the liver is the largest organ in the body and the most exposed to toxins; liver dysfunction may cause various liver diseases (Gordillo et al., 2015). With improvements in living standards, the incidence of metabolic liver diseases such as non-alcoholic and alcoholic fatty liver continues to increase, and alcoholic liver disease further develops into a series of liver diseases and even death (Xiao et al., 2019; Attia et al., 2021). Therefore, it is very essential to prevent the development of ALD in time.

According to literature, ALD pathogenesis includes hepatic steatosis, oxidative stress, and ROS, direct liver toxicity from ethanol metabolism, inflammation induced by cytokines and chemokines, and dysfunction of liver metabolism (Seitz et al., 2018). Steatosis is the earliest response to excessive drinking, which is characterized by fat deposition in liver. This phenomenon is reversible after stopping drinking, but long-term excessive drinking can lead to inflammation, fibrosis, and even cancer (Abo-Salem et al., 2021; Ali et al., 2021; Mohamady et al., 2020). According to statistics, more than 90% of alcoholics show fat accumulation, and 30% of them develop severe ALD (Gala and Vatsalya, 2020). The mechanism behind ALD is complex and not yet fully elucidated. At present, it can be confirmed that excessive ethanol is absorbed by the body, and a large amount of acetaldehyde and Reactive Oxygen Species (ROS) produced in the process of metabolism are important factors leading to liver injury. Excessive ethanol significantly increased the expression of CYP2E1, resulting in the accumulation of acetaldehyde and the production of a large amount of ROS (such as hydrogen peroxide (H2O2), hydroxyl (OH-), and hydroxyethyl (HER)). Excessive accumulation of ROS will lead to oxidative stress damage, lipid peroxidation, DNA mutation, and cell membrane damage (Nagata et al., 2007). Lipid peroxidation is probably the most important response in alcohol-induced liver injury, forming toxic aldehydes, including malondialdehyde (MDA) and 4-hydroxynonenal (4-HNE), which usually lead to changes in the structure, location, and function of proteins (metabolite acetaldehyde has a similar effect), causing severe damage in the liver (Ceni et al., 2014; Galligan et al., 2012; Nagata et al., 2007). The emergence of ROS is closely related to Nrf2. Nrf2 is considered as a liver protective factor. Nrf2 activation can significantly improve the lipid denaturation induced by many factors (such as high-fat diet, alcohol, etc.) (Tanaka et al., 2008). When exposed to electrophiles or ROS, Nrf2 can then be transferred directly to the nucleus, heterodimerize with an sMAF, bind to ARE/EpRE, and strongly activate downstream genes (Adak and Khan, 2019), such as HO-1, NQO1, and other genes (Yamamoto et al., 2018).

In 1998, the concept of “Gut-liver axis” appeared, which explained the relationship between function and two-way interaction between gastrointestinal tract and liver. The gut liver axis refers to the bidirectional relationship between the gut and its microbiota and the liver, which is formed by the integration of signals generated by dietary, genetic, and environmental factors. This interaction is established by the portal vein, which can transport intestinal-derived products directly to the liver and secrete bile and antibodies produced by the liver to the intestine. The destruction of the gut liver axis is manifested by the exposure of microorganisms in the liver, the generation of an oxidizing and pro-inflammatory environment, the destruction of intestinal barriers (including intestinal microbial disorders, intestinal mechanical barriers, weakened chemical barriers, and decreased immune barrier functions), and the increase of portal vein pressure (Albillos et al., 2020).

Under normal circumstances, the structure of the intestinal is stable and its defense function is exerted. However, when the intestinal environment changes, such as inflammation, diet, alcohol, and other factors, the intestinal barrier will be destroyed, resulting in intestinal microbiota and product leakage and transfer to other parts of the body to cause disease. At present, a large number of studies have shown that alcohol and its metabolite acetaldehyde induce oxidative stress, disrupt circadian rhythms, and malnutrition to promote the destruction of intestinal tight junctions (Tang et al., 2008) Portal vein collects and drains from the intestine, nutrients, and metabolites from body and microbiome sources are transported to the liver (Albillos et al., 2020). With this approach, the components of the microbiome may lead to steatohepatitis and fibrosis of the liver (Duan et al., 2019; Schwabe et al., 2006). Intestinal-derived lipopolysaccharide (LPS) from Gram-negative bacteria triggers TLR4-dependent liver injury after intestinal injury (Inokuchi et al., 2011). When the liver senses the increase in LPS, it quickly synthesizes Lipopolysaccharide binding protein (LBP) and secretes it into the circulatory system, which binds to LPS. LBP then expresses LPS to CD14. CD14 specifically binds to LPS and interacts with TLR4 and Myd88 to activate the transcription of downstream nuclear factor NF-kappa B (NF-κB) to regulate the production of inflammatory factors such as TNF- α, IL-1 β, IL-6, TGF-β1, and ROS, and aggravate liver injury (Han et al., 2021b). Therefore, the development of ALD requires a double hit, the first producing steatosis and oxidative stress capable, the second a source of inflammation of initiating significant lipid peroxidation.

This study used network pharmacology was used to predict the possible JGST mechanisms. A total of 323 JGST overlapping targets and ALD were obtained from the databases. Through PPI analysis and KEGG enrichment of the overlapping targets, it was found that the core targets and pathways involved were oxidative stress pathways, fatty acid metabolism, and inflammation pathways. These include NQO1, HO-1, Mammalian target of rapamycin, glutathione metabolism, fatty acid metabolism, and the NF-κB signaling pathway.

According to the records of the Tibetan Medical Masterpiece of “Si Bu Yi Dian”, the causes of liver disease are mainly divided into internal and external causes. Tibetan medicine believes that the external causes are mostly caused by feeling the evil of dampness-heat epidemic toxin, while the internal causes are related to the weakness of the vital qi of the patients. Tibetan medicine believes that an unsuitable diet and daily life will lead to imbalance and disorder of the five sources in the body: Long, Chiba, PeiGen, blood, and Huang water, resulting in “Peigen Mubu”, which causes liver disease (Gyatso, 2010). Tibetan Jia-Ga-Song-Tang (also known as Sanwei Ganjang Powder) is composed of dried ginger, nutmeg, and nutmeg with prescription dosage of 300 g Dried rhizome of Zingiber officinale Roscoe, 200 g Dry ripe fruit of Amomum compactum Sol. Ex Maton and 250 g Dried kernel of Myristica fragrans Houtt. It is a yellowish-brown powder with fragrant gas, and a bitter taste, and is used in the treatment of deficiency-cold hepatitis, hepatomegaly, infantile hepatitis, and so on. The prescription is used in the treatment of liver diseases, and it is included in the “Pharmacopoeia of the People’s Republic of China”. Zingiber officinale Roscoe (Gang Jiang (GJ)), and Amomum compactum Sol. Ex Maton (Dou Kou (DK)), Myristica fragrans Houtt (Rou Doukou (RDK)) are important traditional Chinese medicines with medicinal and food homology. Germacrone is not only a bioactive natural compound found in the traditional medicinal plants of family Zingiberaceae, but also one of the components of nutmeg, so it is selected as a component for cell culture (Dang et al., 2020; Riaz et al., 2020).

As the prescription of JGST contains a large amount of volatile oil, the volatile oil was extracted by steam distillation in the early stage, and the components of the volatile oil were identified by GC-MS. At the same time, the GC-MS results of the total volatile oil of JGST and its single medicine were compared. It was found that the composition and content of the total volatile oil of single medicine and JGST seemed to strengthen the synergism between components. And the change in the direction of reducing the potentially toxic side effects in the single medicine explained the scientific nature and rationality of the compatibility of JGST from the view of a material basis (Tan et al., 2014; Tan et al., 2015). To clarify the protective effect of JGST on liver, the previous studies had observed that JGST can reduce the abnormal increase of liver function enzymes ALT and AST and improve the pathological injury of liver tissue in different liver disease models through acute chemical liver injury model (CCl4, d-galactosamine), acute immune liver injury model (ConA), acute drug-induced liver injury model (APAP), acute alcoholic liver injury model and chronic liver injury model (CCl4). At the same time, it was also observed that JGST has a protective effect on hepatocytes injured by oxidative stress *in vitro*, which shows that JGST has a direct protective effect on hepatocytes (Fu et al., 2014; Liao et al., 2015; Wu et al., 2016; Xiong et al., 2013). Silent the expression of Nrf2 and Bach1 by siRNA *in vitro* also confirmed that JGST played a hepatoprotective effect by promoting Nrf2 into nuclear equilibrium and the exonucleation of Bach1, regulating downstream antioxidant enzymes HO-1 and NQO1, which indicated that JGST balances cellular homeostasis (Chen et al., 2020).

The acute alcoholic model was established by intragastric administration of ethanol in rats. It was found that JGST could reduce the content of MDA and increase the activities of GSH, SOD, and GSH-Px in liver tissue to protect the liver. JGST significantly promoted the expression of Nrf2 and HO-1mRNA, which may be the early treatment of liver injury, it can promote the binding of Nrf2 into the nucleus and antioxidant response of the original ARE. It promotes the expression of antioxidant enzyme HO-1 gene and protein, suggesting that JGST has a certain regulatory effect on the “Nrf2/Bach1-ARE” pathway.

Acute alcohol model can only cause a slight increase of serum transaminase level or hepatocyte steatosis, with mild ballooning and inflammatory cell infiltration and other pathological changes, but can not produce typical human ALD lesions. In the Lieber-Decarli model, steatosis of liver can occur after 4–12 weeks of feeding, and the level of serum transaminase remains unchanged or slightly increased, resulting in mild liver injury, mild liver steatosis, and a small amount of inflammation. Therefore, the establishment of a stable and ideal ALD model is the basis for studying the pathogenesis of ALD. This model is given 8-weeks mice feeding with the 5%v/v Lieber-Decarli alcohol liquid diet. AST and ALT are important biochemical indexes to evaluate liver function. Combined with body weight, liver index, histopathology, and lipid accumulation, it is preliminarily determined that JGST has a protective effect on ALD.

With intake of alcohol, acetaldehyde increases under alcohol dehydrogenase catalysis and produces reactive oxygen species (ROS). However, excessive ROS accumulation may lead to oxidative stress, cell death, lipid peroxidation, DNA and cell membrane damage (Ceni et al., 2014). Furthermore, oxidative stress induced by alcohol and its metabolites disrupts the circadian rhythm and malnutrition, promoting the destruction of tight junctions in the intestine (Zhou and Zhong, 2017). Biochemical kits were used to detect MDA, SOD, LDH, CAT, TG, and TC in the liver and intestine. NQO1, HO-1, GCLC, ZO-1, occludin, FASN, and PPARα were tested by WB or RT-qPCR. In summary, it was found that JGST reduced the production of lipid peroxidation products and improved the antioxidant ability.

There is a state of coexistence of pro-oxidants and antioxidants in chronic alcoholics (Li et al., 2015). We found that JGST down-regulated the antioxidant enzyme in the liver and up-regulated the antioxidant enzyme in the ileum, which may be a compensatory mechanism. In addition, the expression of HO-1 in the liver of patients with non-alcoholic fatty liver also increased significantly, and the increase reflected the severity of the disease (Ignatowicz et al., 2013). The increase of antioxidant enzymes after liver injury may be a compensatory regulatory response to increased oxidative stress (Chen et al., 2011). Compensation is a kind of self-defense mechanism, when the environment changes slightly and has not exceeded the normal regulation function of the human body, the human body adapts to the changing environment through its physiological regulation function, which will not harm the human body at this time. If the environment changes dramatically and exceeds the normal regulatory function of the human body, it will cause abnormal changes in some physiological functions.

Alcohol induces inflammation, and pro-inflammatory cytokines, such as TNF can promote ROS production and further aggravate liver damage. This study detected the expression of CD14 and Myd88 inflammatory proteins, and the levels of IL-1β, TNF-α, IL-6, and TGF-β1 were detected in this study. Thus, they were collectively showing that JGST has an inhibitory effect on inflammation induced by alcohol, which is also in line with the high degree of TNF in the PPI analysis in the previous network pharmacology.

The intestinal barrier damage leads to LPS leakage. Therefore, LPS is released into the blood and enters the liver via the hepatic portal vein, causing a two-hit to the liver (Tsutsui and Nishiguchi, 2014; Yang et al., 2019). ELISA kits were used to detect the changes in the LPS and LBP levels in the mice plasma. Finally, JGST was found to protect the intestinal barrier and prevent LPS leakage into the blood.

Lipid peroxidation may be the most important reaction in alcohol-induced liver injury. This results in toxic aldehydes generation, including malondialdehyde (MDA) and 4-hydroxynonenal (4-HNE), which usually leads to changes in protein structure, localization, function, and activity; ethanol metabolism of acetaldehyde also has a similar effect, causing serious damage to the liver (Galligan et al., 2012). In the differential protein analysis of mouse liver in proteomics, it was found that JGST affects ALD in lipid peroxidation, oxidative stress, fatty acid *ß* oxidation, and inflammation.

At the cellular level, PCR and western blot results showed that the mRNA and protein levels of HO-1 and NQO1 were significantly balanced in the ethanol-induced ALD model cells compared with the control group, suggesting that the stimulation of alcohol will result in imbalance of the Nrf2. In the ALD model of HepG2 cells, the expression of HO-1 and NQO1 protein and mRNA was mainly increased. After the treatment with Germacrone, the expressions of Nrf2-related factors tended to return to homeostasis. Germacrone can significantly increase the expression of ZO-1 and Occludin mRNA to alleviate the ethanol-induced injury of intestinal barrier to protect the liver from Two-hit in IEC-6. JGST can effectively alleviate ALD by regulating Gut-liver axis and cellular homeostasis.

Among the differentially expressed proteins in proteomics is nicotinamide N-methyltransferase (NNMT), an intracellular methyltransferase, which is expressed in large amounts in the liver and is considered to be a cell metabolism and energy regulator (Song et al., 2020; Xu et al., 2021). Studies have shown that inhibiting NNMT can protect the liver from lipotoxicity, further elucidating its potential clinical application in metabolic diseases (Alexandra Griffiths et al., 2021). Another example is hydroxysteroid 17-β dehydrogenase 13 (HSD17B13), which is mainly expressed in the liver. It encodes liver lipid droplets and plays a role in lipid metabolism. Studies have shown that HSD17B13 may regulate the HSCs activity and participate in the development of liver fibrosis (Ma et al., 2019). The application of proteomics could predict and indicate the follow-up research, and proteomics will become one of the most effective methods to find disease molecular markers and drug targets (Chahrour et al., 2015; Gibriel, 2012; Souissi et al., 2021).

**FIGURE 9:**
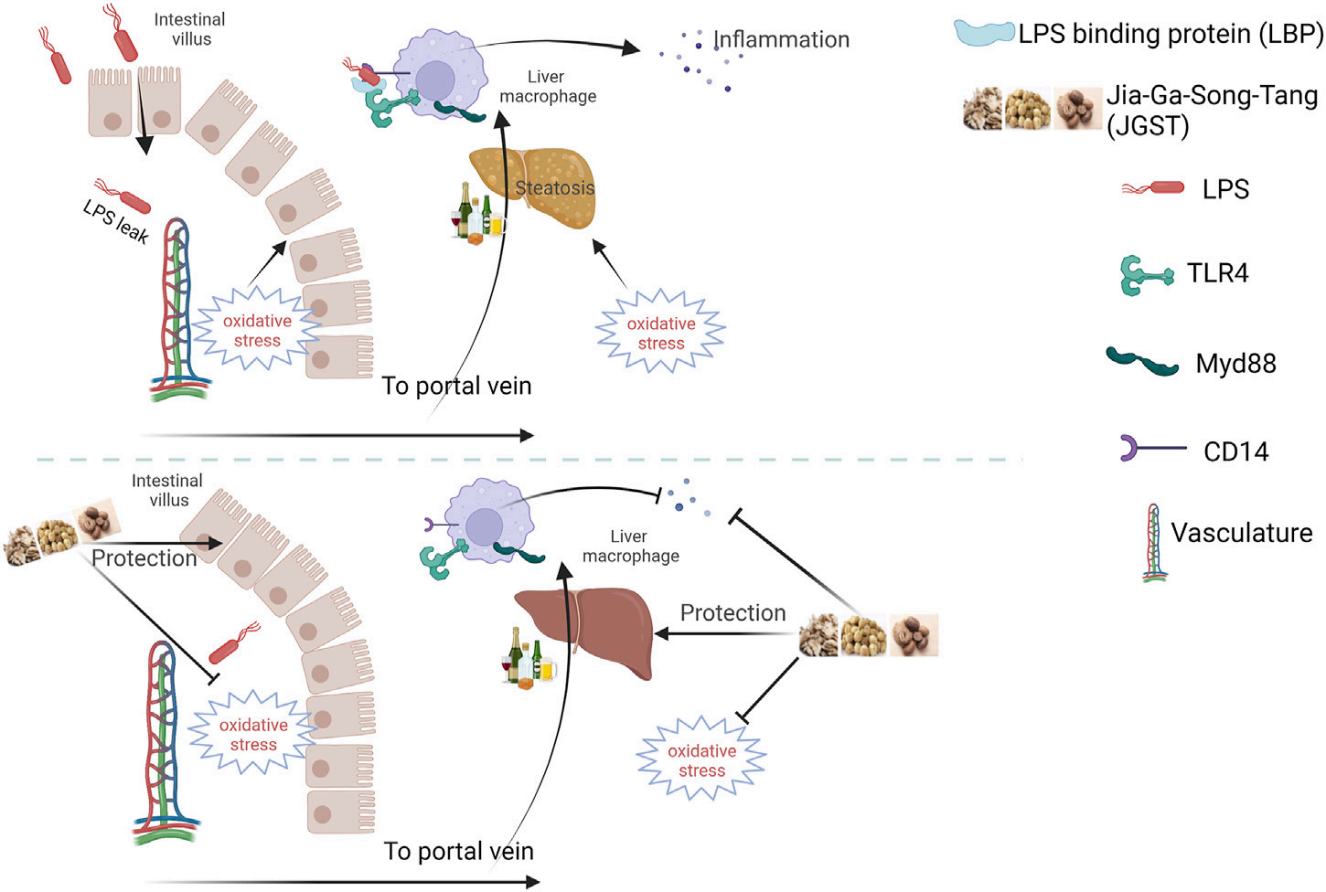
Schematic diagram of Jia-Ga-Song-Tang protection against alcoholic liver and intestinal damage. It was created in the website www.BioRender.com.

In summary, JGST showed excellent protection against alcohol-induced damage by regulating Gut-liver axis and cellular homeostasis. The underlying mechanism may be to reduce oxidative stress and lipid accumulation, thereby inhibiting liver inflammation and reducing pathological injury. Furthermore, it can repair the intestinal barrier to protect the liver from two hits (Figure 9). However, the mechanism of lipid metabolism derived from proteomics will be further revealed in future studies.

## Conclusion

The study preliminarily clarified that JGST, which contains three traditional Chinese herbs with dual officinal and edible functions, can improve alcohol-induced liver and intestinal injury, mainly by regulating Gut-liver axis and cellular homeostasis, improving liver steatosis and the intestinal barrier. It has also been proven that JGST has a certain protective effect on intestinal injury and reduces the translocation of LPS to prevent a two-hit to the liver. Therefore, further studies are necessary to determine the underlying mechanism.

## Data Availability

The datasets presented in this study can be found in online repositories. The names of the repository/repositories and accession number can be found below and in the Supplementary Material: iProX (http://www.iprox.org), accession IPX0004632000.

## Glossary

ALD: alcoholic liver damage
ALT: alanine transaminase
AST: Aspartate aminotransferase
Bach1: Transcription regulator protein
CAT: catalase
CD14: Monocyte differentiation antigen CD14;
DisGeNET: a database of gene-disease associations
DRUGBANK: Open Data Drug&Drug Target Database
FASN: Fatty acid synthase
GCLC: Glutamate-Cysteine Ligase Catalytic
GC-MS: Gas chromatography-mass spectrometry;
GeneCards: The Human Gene Database
GO: gene ontology
GSH: glutathione
GSH-Px: Glutathione peroxidase
HE: Haematoxylin-eosin
Herb: A high-throughput experiment- and reference-guided database of traditional Chinese medicine.
HO-1: Heme oxygenase-1
HPLC: high-performance liquid chromatography
IL-1β: interleukin-1β
IL-6: interleukin-6
KEGG: Kyoto Encyclopedia of Genes and Genomes
LBP: lipopolysaccharide-binding protein
LDH: lactate dehydrogenase
LPS: lipopolysaccharide
MDA: malondialdehyde
Myd88: myeloiddifferentiationfactor88
NQO1: NAD(H)P-quinone-oxidoreductase -1
Nrf2: Nuclear factor erythroid 2-related factor 2
OCT: opti-mum cutting temperature compound
OMIM: Online Mendelian Inheritance in Man
PPARα: Peroxisome proliferator-activated receptors-alpha
PPI: the Protein-protein interaction
Pubchem: the world’s largest collection of freely accessible chemical information
PVDF: polyvinylidene fluoride
ROS: reactive oxygen species
SOD: superoxide dismutase
SRTING: Search Tool for the Retrieval of Interacting Genes
Swiss Target Prediction: a web server for target prediction of bioactive small molecules
TC: total cholesterol
TG: triglyceride
TGF-β1: transforming growth factor-β1
TNF-α: tumor necrosis factor-α
TTD: Therapeutic Target Database
Two-hit: ‘two-hit’ hypothesis
ZO-1: Zonula occludens-1
