# Supplementary material for: Jia-ga-song-tang protection against alcoholic liver and intestinal damage
Source: Front Pharmacol. 2022 Sep 26;13:981706. doi: 10.3389/fphar.2022.981706 (PMC9549243; doi:10.3389/fphar.2022.981706)
Supplement: Supplementary file 1 [file DataSheet1.pdf]

Supplementary Material

1 Supplementary Data

Supplementary Methods

1. Immunofluorescence analysis

The Hepg2 were inoculated into a petri dish with pre-treated cover slides. When the Hepg2 were close to growing into a monolayer, the cover slides were removed and washed twice by PBS. After fixing with cross-linking agents (paraformaldehyde) generally need to be permeated before incubating with antibodies to ensure that antibodies can reach the antigen site. The time of penetration is 15min. Followed by blocking with 10 % goat serum for 30 min at room temperature. Next, the Hepg2 were incubated with anti-Nrf2 antibodies (Affinity Biosciences LTD, Jiangsu, China) overnight at 4 ℃. After through washing, cells were further stained with anti-rat fluorescentsecondary antibodies (Proteintech Group, Inc., Wuhan, China) for 1h at room temperature. Finally, 4′ ,6-diamidino-2-phenylindole (DAPI) (Solarbio Science&Technology Co.,Ltd, Beijing, China) was used for nuclei staining. Images were captured using Olympus microscope (Olympus Co., Ltd.Zeiss, China) and viewed.

2 Supplementary Figures and Tables

2.1 Supplementary Figures

Figure Legends

Supplementary Figure 1. Protective effect of JGST against alcohol-induced liver injury in HepG2 involved modulation of Nrf2. (A) The protein expression of Nrf2 in HepG2 (n=4). (B) Immunofluorescence staining for Nrf2. (400× original magnification, n=3). Control group, Model group, ML385: ML385 group with alcohol, GM-L: Germacrone (0.5 μM) group with alcohol, GM-M: Germacrone (1.0 μM) group with alcohol, TBHQ: TBHQ group with alcohol, TBHQ+L: TBHQ and Germacrone (0.5 μM) group with alcohol, TBHQ+M: TBHQ and Germacrone (1.0 μM) group with alcohol. Data were presented as Mean±SEM, Compared with Control group, $^{*}P<0.05$, $^{**}P<0.01$, $^{***}P<0.001$.

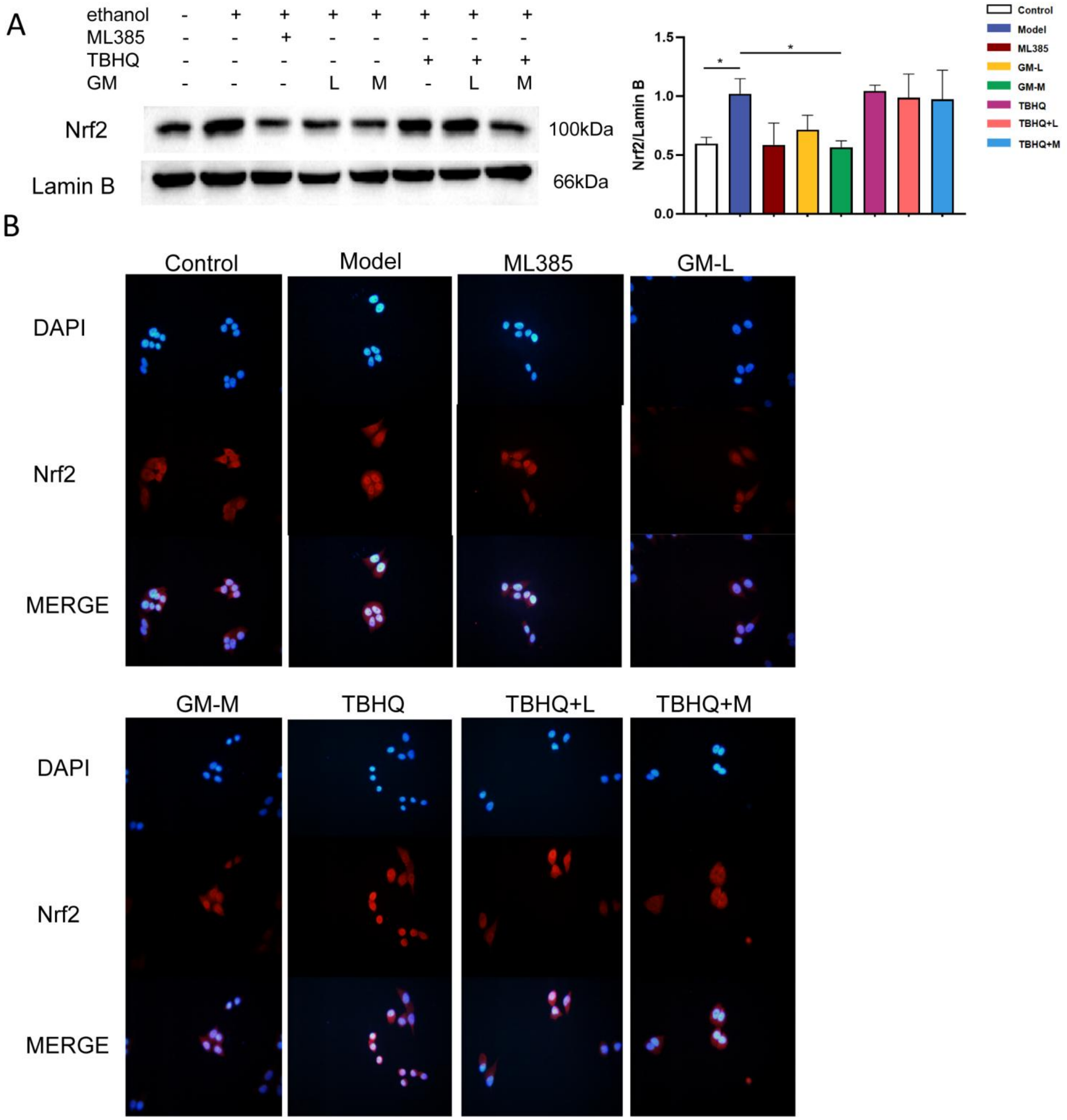

Supplementary Figure 1. Protective effect of JGST against alcohol-induced liver injury in HepG2 involved modulation of Nrf2.

2.2 Supplementary Tables

Supplementary Table1. Antibodies and Dilution ratio

Antibody	Vendor	Catalog number	Dilution ratio
HO-1 antibody	Abcam Trading (Shanghai) Co., Ltd.	ab13243	1:2000
NQO1 antibody	Affinity Biosciences LTD	DF6437	1:2000
GAPDH antibody	Affinity Biosciences LTD	AF7021	1:5000
NRF2 antibody	Affinity Biosciences LTD	AF0639	1:2000
Beta-actin antibody	Affinity Biosciences LTD	AF7018	1:5000
Lamin B antibody	Affinity Biosciences LTD	AF5161	1:2000
CD14 antibody	Cell Signaling Technology	93882	1:1000
Myd88 antibody	Cell Signaling Technology	4283s	1:1000
HRP-conjugated Affinipure Goat Anti-Rabbit IgG(H+L)	ProteinTech Group	SA00001-2	1:5000
CoraLite594-conjugated Goat Anti-Rabbit IgG(H+L)	ProteinTech Group	SA00013-4	1:200

Supplementary Table2. Materials and reagents

Medicine/reagents	Vendor	Catalog Number
Gang Jiang (GJ)	Guangdong Province Traditional Chinese Medicine Hospital	YPB0G0001
Dou Kou (DK)	Guangdong Province Traditional Chinese Medicine Hospital	200501
Rou Doukou (RDK)	Guangdong Province Traditional Chinese Medicine Hospital	200401

Silymarin	MADAUS GMBH, Germany	B1902304
Germacrone	Shanghai Yuanye Bio-Technology Co.,Ltd	B20589
ML385	MedChemExpress	HY-100523
tert-Butylhydroquinone (TBHQ)	MedChemExpress	HY-100489
Ethanol (95%)	Shanghai Aladdin Biochemical Technology Co.,Ltd.	B20122024
Ethanol (≥99.7%)	Sinopharm Chemical Reagent Co.Ltd.	100092683
HE solution	Wuhan Servicebio Technology CO.,LTD	G1005
Oil Red O solution	Beijing Solarbio Science&Technology Co.,Ltd.	G1260
Alanine aminotransferase Assay Kit	Nanjing Jiancheng Biological Engineering Research Institute	C009-2-1
Aspartate aminotransferase Assay Kit	Nanjing Jiancheng Biological Engineering Research Institute	C010-2-1
Malondialdehyde (mda) assay kit	Nanjing Jiancheng Biological Engineering Research Institute	A003-1-2
Superoxide Dismutase (sod) assay kit	Nanjing Jiancheng Biological Engineering Research Institute	A001-3-2
catalase (cat) assay kit	Nanjing Jiancheng Biological Engineering Research Institute	A007-2-1
Reduced glutathione (GSH) assay kit	Nanjing Jiancheng Biological Engineering Research Institute	A006-2-1
Triglyceride assay kit	Nanjing Jiancheng Biological	A110-1-1

	Engineering Research Institute	
Total cholesterol assay kit	Nanjing Jiancheng Biological Engineering Research Institute	A111-1-1
RNAeasy™ Animal RNA Isolation Kit with Spin Column	Beyotime Biotech Inc	R0027
PrimeScript™ RT Master Mix (Perfect Real Time)	TaKaRa Biomedical Technology(Beijing)Co.,Ltd.	AK22353A
TB Green® Premix Ex Taq™ II (Tli RNaseH Plus)	TaKaRa Biomedical Technology(Beijing)Co.,Ltd.	AJE1687A
Xylene	Sinopharm Chemical Reagent Co.Ltd.	10023418
Optimal Cutting Temperature compound	Beijing Solarbio Science&Technology Co.,Ltd	G1005-3
Glycerol Gelatin aqueous slide mounting medium	Beijing Solarbio Science&Technology Co.,Ltd	S2150
ELISA Kit for Lipopolysaccharide Binding Protein (LBP)	Cloud-clone Corp.wuhan	SEB406Mu
ELISA Kit for Lipopolysaccharide (LPS)	Cloud-clone Corp.wuhan	HEB526Ge
IL-1 beta Mouse Uncoated ELISA Kit	Thermo Fisher Scientific	88-7013
Olympus microscope	Olympus Co., Ltd.Zeiss	BX53
Multiskan SkyHigh	Thermo Fisher Scientific	A51119500C
NanoDrop Lite	Thermo Fisher Scientific	Nano Drop 2000
Quantitative Real-time PCR	Thermo Fisher Scientific	ABI7500

Electrophoresis and blotting system	Bio-Rad Laboratories, Inc.	1658030
Carbon dioxide incubator	Esco Micro Pte. Ltd.	CLM-170B-8-NF
